# Takayasu Arteritis Following Necrotizing Fasciitis: A Case Report and Brief Review of the Literature

**DOI:** 10.7759/cureus.90427

**Published:** 2025-08-18

**Authors:** Maria J Lizarazo Jimenez, Laura Campaña Perilla, Carlos M Olarte Salazar, Eduardo A Iregui Cantor

**Affiliations:** 1 Internal Medicine, University Hospital Fundación Santa Fe de Bogotá, Bogota, COL; 2 Radiology, University Hospital Fundación Santa Fe de Bogotá, Bogota, COL; 3 Traumatology, University Hospital Fundación Santa Fe de Bogotá, Bogota, COL

**Keywords:** infection, large-vessel vasculitis, necrotizing fasciitis, takayasu arteritis, vasculitis

## Abstract

Takayasu arteritis (TA) is a rare, immune-mediated large-vessel vasculitis that primarily affects the aorta and its major branches. While several infectious agents have been implicated as potential environmental triggers, necrotizing fasciitis (NF) has not been previously linked to the onset of TA. We report the case of a previously healthy 33-year-old woman who developed rapidly progressive TA following NF of the left lower limb. Despite broad-spectrum antibiotics and surgical debridement, the patient exhibited persistent fever, leukocytosis, and new-onset hypertension. Imaging revealed new inflammatory changes in the thoracic and abdominal aorta, consistent with large-vessel vasculitis. A diagnosis of TA was established based on the findings of magnetic resonance angiography. Following high-dose corticosteroid therapy, the patient showed rapid clinical improvement. Cyclophosphamide was initiated, and follow-up imaging confirmed partial resolution of vascular inflammation. This case highlights NF as a potential immunologic trigger for TA. In patients with persistent systemic inflammation despite adequate infection control, autoimmune vasculitis should be considered as a possible underlying cause. Early diagnosis and prompt immunosuppressive therapy can lead to significant clinical improvement.

## Introduction

Takayasu arteritis (TA) is a chronic large-vessel vasculitis that primarily affects the aorta and its major branches [1]. It has a reported prevalence ranging from 3.2 to 40.0 cases per million and an incidence between 0.4 and 2.6 cases per million annually [2]. The clinical course of TA is usually biphasic. An initial systemic phase may present nonspecific symptoms such as low-grade fever, fatigue, weight loss, and arthralgias [3,4]. As the disease progresses, vascular involvement becomes evident, often presenting with diminished or asymmetric pulses, limb claudication, blood pressure discrepancies, and arterial bruits [4]. Renal artery stenosis and reduced aortic compliance frequently contribute to hypertension [3]. Laboratory findings are nonspecific but typically reflect systemic inflammation, including elevated erythrocyte sedimentation rate (ESR), C-reactive protein (CRP), leukocytosis, normocytic anemia, and thrombocytosis. Notably, normal inflammatory markers do not exclude disease activity [3].

While the pathogenesis of TA remains incompletely understood, it is widely accepted to be immune-mediated [5]. A growing body of literature suggests that infectious exposures may act as environmental triggers rather than primary causes of disease. Mycobacterium tuberculosis has been the most frequently associated pathogen, with proposed mechanisms involving chronic immune activation and molecular mimicry. Several case series and observational studies report a higher prevalence of latent or active tuberculosis among TA patients, especially in endemic regions [6]. Beyond M. tuberculosis, case reports have implicated other infectious agents, including Streptococcus pyogenes, Varicella-zoster virus, Hepatitis B virus, HIV, and, more recently, SARS-CoV-2 [7-11]. These associations are essentially temporal and vary in strength. Still, they support the hypothesis that certain infections may precipitate or amplify vascular inflammation in genetically susceptible individuals [5]. In line with this hypothesis, we present a case of rapidly progressive TA in which necrotizing fasciitis (NF) appeared to act as a proinflammatory infectious trigger.

## Case presentation

A previously healthy 33-year-old female patient presented to the emergency room with a four-day history of nausea, abdominal pain, and fever after sustaining blunt trauma to the left foot and leg. Physical examination of the left lower limb revealed edema and phlyctenae, without other signs of infection. A basic workup revealed leukocytosis and neutrophilia. A skin and soft tissue infection was suspected, and wound cultures were obtained. Empirically, vancomycin plus ceftriaxone was started. The left lower limb ultrasound showed an infra-malleolar collection.

Early in her admission, she developed worsening leukocytosis and hypotension requiring vasopressor support and transfer to the ICU. An orthopedic consult was requested, and she was taken for emergent surgery for suspicion of NF, where fatty liquefaction and abundant purulent secretion were found. Tissue cultures were negative after 5 days, and after three surgical lavages of the area, the patient was persistently febrile and had now become hypertensive. On day 12, she developed worsening tachycardia, desaturation, and retrosternal pain. Cardiac markers were positive, with a negative troponin delta and no electrocardiographic changes; therefore, pulmonary thromboembolism was ruled out with a chest CT. Upon further questioning, the patient reported a history of multiple complicated infections, so a study of primary immunodeficiencies was performed, which revealed hypogammaglobulinemia of the G and M types, along with typical autoantibodies. A dose of immunoglobulin G was administered, which momentarily reduced the patient's leukocytosis.

The patient required invasive ventilatory support with a subsequent episode of pulseless electrical activity for which a successful cardiovascular resuscitation protocol was performed. An echocardiogram showed an ejection fraction of 28%, considering cardiac arrest secondary to cardiogenic shock due to stress cardiomyopathy secondary to septic shock. At this point, the patient had seven sets of negative blood cultures and five negative intraoperative cultures. During her ICU stay, she received multiple antibiotic combinations, including piperacillin/tazobactam plus linezolid, ceftazidime avibactam plus aztreonam with linezolid, and amphotericin B without improvement. Despite aggressive source control with five surgical lavages of the area on day 23 of admission, transfemoral amputation of her left lower limb was performed.

**Figure 1:**
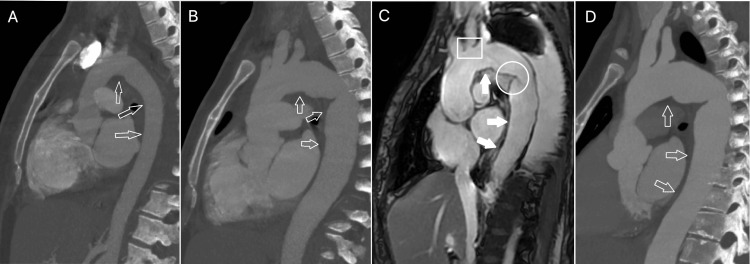
Evolution of aortic involvement in Takayasu arteritis. (A) Sagittal thoracic CT angiography (bone window) showing standard aortic caliber (arrows). (B) Repeat CT angiography 22 days later, demonstrating diffuse thickening of the aortic arch, including both ascending and descending segments (arrows). (C) Chest magnetic resonance angiography (MRA) confirming concentric wall thickening of the thoracic aorta. A square highlights stenosis of the ostium of the right common carotid artery, and a circle denotes an intimal flap in the distal thoracic aorta. (D) Follow-up CT angiography performed six weeks later shows moderate improvement in aortic thickening.

Given her persistent fever with leukocytosis in what appeared to be adequate source control and the absence of other sources suggestive of infection a new search for non-infectious causes of fever was performed which included a repeat basic work-up for autoimmune diseases which was negative and a CT angiography of the chest that revealed inflammatory changes of the aorta not present in the chest CT performed 22 days before (Figures 1A, 1B). A thoracic chest magnetic resonance angiography was performed to evaluate the possibility of large- and medium-vessel vasculitis, such as TA. The MRI confirmed the findings, showing a slightly dilated thoracic and abdominal aorta in its ascending and descending portions (Figure 1C). There was also evidence of stenosis of the ostium of the right carotid artery and intimal flap in the descending third of the aorta (Figure 1C). Based on these findings, the patient met diagnostic criteria for TA, and high-dose methylprednisolone pulses were initiated.

Following the initiation of corticosteroid therapy, the patient experienced a marked clinical improvement, including normalization of heart rate, resolution of hypertension, and cessation of febrile episodes. A decrease in leukocytosis, neutrophilia, and serum lactate levels paralleled these changes. The patient was discharged with plans to initiate cyclophosphamide, alongside outpatient follow-up with hematology for management of her hypogammaglobulinemia and rheumatology for continued monitoring of TA. A repeat chest CT angiography performed one month later demonstrated modest improvement in the previously noted aortitis (Figure 1D).

## Discussion

The diagnosis of TA in our patient was based on *The European League Against Rheumatism* (EULAR)/*American College of Rheumatology* (ACR) 2022 classification criteria, which require an age at diagnosis of 60 years or younger and imaging evidence of vasculitis as mandatory entry criteria. In addition, our patient met several weighted clinical and imaging features: she experienced ischemic chest pain (angina), had imaging-confirmed carotid artery abnormalities, and showed involvement of multiple arterial territories on vascular imaging [12]. These findings met the minimum score required for classification, thereby supporting the diagnosis of TA in the context of persistent systemic inflammation and negative infectious workup.

The clinical presentation of our patient did not follow the typical indolent course of TA, which usually develops over months with nonspecific symptoms such as fatigue and low-grade fever, often delaying diagnosis [3]. In contrast, she exhibited marked systemic inflammation and new-onset hypertension within days of the NF. The persistence of fever, leukocytosis, and hypertension despite adequate source control and antibiotics raised suspicion for vasculitis. Leukocytosis is reported in approximately 44% of cases of TA, and constitutional symptoms like fever and malaise are frequent at onset [13]. Additionally, hypertension, often related to renal or aortic involvement, occurs in 30% to 80% of patients with TA [14]. This atypical and rapidly progressive course, especially in the setting of recent NF, prompted early evaluation for vasculitis and consideration of TA.

Proposed pathogenic mechanisms include aberrant immune responses to vascular injury, dysregulated T-cell activation, and chronic inflammatory stimuli that may be triggered by prior infections [15]. A growing body of evidence suggests that infectious agents may serve as the external stimuli that breach this vascular immune privilege and trigger pathologic inflammation. Among the proposed triggers of this aberrant immune activation, infections, particularly those caused by Mycobacterium tuberculosis, have received considerable attention, possibly through mechanisms involving molecular mimicry and dysregulated immune responses [16]. While *M. tuberculosis* remains the most consistently associated pathogen, other microorganisms, such as Streptococcus species, have also been investigated. Elevated antistreptolysin O (ASO) titers have been reported in patients with TA, suggesting a possible immunologic link, but no causal relationship has been definitively established, and the evidence remains inconclusive [7]. Moreover, infections are recognized triggers of autoimmune and hyperinflammatory responses, raising the possibility that severe bacterial infections could initiate or exacerbate autoimmune vasculitis [15]. Necrotizing fasciitis, a fulminant soft tissue infection most commonly caused by *Streptococcus pyogenes*, *Staphylococcus aureus*, or polymicrobial flora including anaerobes and Gram-negative bacilli, induces a profound systemic inflammatory response [17]. Although not previously associated with TA, NF may similarly act as a potent immunologic trigger capable of breaching vascular tolerance in susceptible individuals.

This pattern of infectious events preceding vasculitis is not unique. Both acute and chronic illnesses, including those caused by viral, bacterial, and mycobacterial agents, have been temporally associated with disease onset or relapses. Table 1 presents cases in which TA developed following a defined infectious or immune-related event.

**Table 1:** Summary of published cases of Takayasu arteritis following acute or chronic infections and vaccinations. MMF, mycophenolate mofetil

Author (Year)	Patient(s) (Age and sex)	Trigger	Interval trigger to TA	Clinical presentation	Treatment/Outcome
Acute Infections					
Gilden et al. (2016) [8]	12 autopsy cases, 32-83 years old, 7 females and 5 males	Varicella-zoster virus (VZV)	Variable	Granulomatous aortitis; VZV DNA in the vessel wall	-
Lloyd et al. (2014) [7]	19-years-old Female	Post-streptococcal pharyngitis	4 months	Aortic dilatation and regurgitation	Steroids + MMF; significant improvement
Chronic infections					
Ferfar et al. (2018) [10]	7 patients 36-56-year-old, 4 females and 3 males	HIV infection	Not specified	Aortic and supra-aortic involvement, systemic symptoms, ↑IL-6/IFN-γ	Steroids ± immunosuppressants; frequent relapse or poor response.
Ozenirler et al. (2007) [9]	26-year-old female	Chronic hepatitis B (HBV)	Chronic	Pulseless upper limb	Antivirals + methotrexate; clinical stability
Miller and Friedman (2004) [18]	27-year-old female	Chronic hepatitis C (HCV)	3 years (symptom evolution)	Fever, hypertension, subclavian, and carotid stenosis	prednisone + azathioprine; clinical stability
Magar et al. (2021) [16]	5- to 75-year-old; mostly female	Mycobacterium tuberculosis	Simultaneous or 1-6 months delay	Fever, weight loss, limb claudication, pulse deficit, hypertension, chest pain, cough	Anti-TB treatment ± steroids
Liebscher et al. (2018) [19]	56-year-old female	Chronic HBV + latent tuberculosis	12 months (symptom evolution)	Syncope, chest pain, pulmonary artery stenosis.	Prednisone + azathioprine+ methotrexate; stabilization
Vaccination-associated					
Katsouli et al. (2023) [11]	52-year-old female	AstraZeneca COVID-19 vaccine	2 weeks post-second dose	Persistent fever; concentric aortic thickening	IV steroids + cyclophosphamide; remission

Finally, the patient’s rapid clinical improvement following corticosteroid initiation, marked by resolution of fever, normalization of heart rate and blood pressure, and a decline in leukocytosis and lactate levels, supports the diagnosis of active TA and highlights the central role of prompt immunosuppressive therapy. Current guidelines from the EULAR and ACR recommend high-dose glucocorticoids as first-line treatment for active disease, with the addition of steroid-sparing agents such as cyclophosphamide or methotrexate in cases of severe, refractory, or relapsing disease [20]. In our patient, the decision to initiate cyclophosphamide was guided by the severity of vascular inflammation and the need for durable immunosuppression. Follow-up imaging with chest CT angiography one month later demonstrated a modest improvement in aortitis, consistent with the expected radiographic response under guideline-based therapy.

## Conclusions

TA is a rare and often underdiagnosed large-vessel vasculitis that typically follows a subacute course, with nonspecific symptoms contributing to significant diagnostic delays. This case illustrates a rare presentation in which TA developed rapidly following NF, a fulminant soft tissue infection not previously associated with this vasculitis. The patient’s clinical trajectory, marked by persistent fever, leukocytosis, and new-onset hypertension despite effective source control and broad-spectrum antibiotics, highlights the importance of considering autoimmune etiologies in critically ill patients with an unresolved inflammatory state. Imaging findings, in conjunction with the patient’s favorable response to immunosuppressive therapy, supported the diagnosis of TA. This underscores the value of early multimodal assessment, including vascular imaging and clinical suspicion, particularly in atypical settings. Clinicians should remain vigilant about the possibility that severe infections may act as immunologic triggers for vasculitis in individuals predisposed to the condition.
